# Prevention of cisplatin‐induced hearing loss in children: Informing the design of future clinical trials

**DOI:** 10.1002/cam4.1563

**Published:** 2018-05-30

**Authors:** Lori M. Minasian, A. Lindsay Frazier, Lillian Sung, Ann O’Mara, Joseph Kelaghan, Kay W. Chang, Mark Krailo, Brad H. Pollock, Gregory Reaman, David R. Freyer

**Affiliations:** ^1^ National Cancer Institute Bethesda MD USA; ^2^ Dana‐Farber Cancer Institute/Boston Children’s Hospital Cancer Center Boston MA USA; ^3^ The Hospital for Sick Children Toronto ON Canada; ^4^ Department of Otolaryngology Stanford University Palo Alto CA USA; ^5^ Department of Preventive Medicine Keck School of Medicine University of Southern California Los Angeles CA USA; ^6^ Department of Public Health Sciences University of California Davis CA USA; ^7^ US Food and Drug Administration Silver Spring MD USA; ^8^ Division of Hematology, Oncology, and Blood and Bone Marrow Transplantation Children’s Hospital Los Angeles Keck School of Medicine University of Southern California Los Angeles CA USA

**Keywords:** cisplatin, hearing loss, pediatric, prevention, study design

## Abstract

Cisplatin is an essential chemotherapeutic agent in the treatment of many pediatric cancers. Unfortunately, cisplatin‐induced hearing loss (CIHL) is a common, clinically significant side effect with life‐long ramifications, particularly for young children. ACCL05C1 and ACCL0431 are two recently completed Children’s Oncology Group studies focused on the measurement and prevention of CIHL. The purpose of this paper was to gain insights from ACCL05C1 and ACCL0431, the first published cooperative group studies dedicated solely to CIHL, to inform the design of future pediatric otoprotection trials. Use of otoprotective agents is an attractive strategy for preventing CIHL, but their successful development must overcome a unique constellation of methodological challenges related to translating preclinical research into clinical trials that are feasible, evaluate practical interventions, and limit risk. Issues particularly important for children include use of appropriate methods for hearing assessment and CIHL severity grading, and use of trial designs that are well‐informed by preclinical models and suitable for relatively small sample sizes. Increasing interest has made available new funding opportunities for expanding this urgently needed research.

## 1 INTRODUCTION

Cisplatin is an essential chemotherapeutic agent in the treatment of neuroblastoma, osteosarcoma, hepatoblastoma, germ cell tumors, medulloblastoma, and other pediatric cancers.^1^ Unfortunately, cisplatin commonly causes sensorineural hearing loss that is bilateral, irreversible, and may progress over time.^2^ Cisplatin‐induced hearing loss (CIHL) affects all ages. However, in children, especially very young children, it is particularly debilitating because hearing loss leads to impaired language acquisition, difficulty with learning and psychological development, and subsequent reduction in social functioning that will affect them for the remainder of their lives.^3^, ^4^ Long‐term studies of pediatric cancer survivors demonstrate that CIHL results in lower educational performance, increased need for special education services, higher unemployment and nonindependent living, and poorer child‐reported quality of life compared with controls.^5^, ^6^, ^7^ Depending on the cumulative dose and the dosing schedule, as many as 75% of children with CIHL qualify for hearing aids or hearing assistance.^3^, ^4^, ^8^ However, survivors with hearing aids still experience abnormal hearing, tinnitus, poor speech discrimination in noisy environments, social hardships, and substantial expense.^9^, ^10^

Because of these serious consequences, there is increasing interest in identifying effective strategies for preventing CIHL. Historically, preventing CIHL has been limited to cisplatin dose reduction or deletion, with or without substitution by its somewhat less ototoxic analog, carboplatin. However, cisplatin dose modifications are protocol specific and variable.^11^, ^12^ Further, reducing cisplatin dose intensity may jeopardize treatment efficacy, and carboplatin is not uniformly equally effective.^13^ Dose reductions for CIHL are typically triggered after significant hearing loss has occurred, which is particularly important as hearing loss may worsen after continued exposure and completion of cisplatin.^14^ An alternative approach to preventing CIHL is use of otoprotective agents. Conceptually, a successful otoprotective agent would protect hearing without compromising chemotherapy efficacy. A recent systematic review evaluated multiple potential otoprotectants including amifostine, glutathione, calcium, and magnesium, but none demonstrated efficacy in randomized clinical trials.^15^

The Children’s Oncology Group (COG) Cancer Control and Supportive Care Committee develops studies to reduce treatment‐related toxicity among children with cancer.^16^ The COG recently completed two complementary studies addressing CIHL: ACCL05C1, an observational study to compare alternative methods for measuring CIHL;^17^ and ACCL0431, a randomized trial to evaluate sodium thiosulfate (STS) for prevention of CIHL.^18^ To our knowledge, these are the first published cooperative oncology group trials focused solely on CIHL. Given the importance and challenging nature of otoprotection research, the purpose of this paper was to describe issues that emerged from conducting these two studies (ACCL05C1 and ACCL0431) to inform the optimal design of future clinical trials evaluating agents for the prevention of CIHL.

## 2 CHALLENGES IN PREVENTING CIHL

Research methodology for evaluating new agents to prevent CIHL is fraught with challenges. These include identifying otoprotective agents that do not interfere with the anticancer effects of the chemotherapy and are safe for patients; utilizing appropriate preclinical models that optimize translation of the agent into human trials; and designing pediatric clinical trials that can be conducted with reasonable sample sizes, are expected to accrue satisfactorily and are practical.

### 2.1 Mechanisms and modifiers of CIHL

The pathophysiology of CIHL must be considered in designing otoprotection studies. Factors associated with CIHL include age <5 years at time of treatment,^19^, ^20^ cumulative cisplatin dose, duration of cisplatin infusion,^21^ cranial irradiation,^8^, ^22^, ^23^ concomitant exposure to loop diuretics and aminoglycosides,^4^, ^24^, ^25^ and underlying genetic susceptibility.^26^, ^27^, ^28^, ^29^ Carboplatin also can cause hearing loss, especially at high doses or in very young children.^30^

Mechanisms underlying development of CIHL are complex, and knowledge about them is evolving. In brief, cisplatin crosses the blood‐labyrinth barrier to gain access to cochlear tissues.^31^ Whereas the primary mechanism of cisplatin’s anticancer activity is disruption of DNA replication and repair,^32^, ^33^ CIHL is due principally to generation of reactive oxygen species that induce mitochondrial damage and apoptosis of the cochlear outer hair cells.^34^, ^35^, ^36^ Pools of protective natural antioxidants such as glutathione are depleted.^37^ Cisplatin also appears to damage the stria vascularis and reparative stem cells of the inner ear,^38^ resulting in increased cochlear uptake of cisplatin in the face of compromised cellular rescue. Further delineation of the effects of ototoxic and otoprotective agents on the supporting cells, neurons, and central auditory pathways is needed to clarify mechanisms beyond cochlear hair cell loss and to inform targeted interventions.^39^

### 2.2 Preclinical models for identifying otoprotective agents

Guinea pig, rat, hamster, and gerbil models have been used for screening agents for cisplatin otoprotection. Species‐specific differences in organ tolerance require various cisplatin doses, schedules, and routes of administration. Recently, the zebrafish has emerged as a novel model for this purpose.^40^ Agents showing otoprotection must be screened for potential interference with cisplatin chemotherapy effects. This may be performed using in vitro cancer cell cytotoxicity assays, but generally requires tumor xenograft studies using nude mouse models. Thus, preclinical research for otoprotective agents is complex in that a single animal model cannot be used for both otoprotection and tumor protection screening.

The goal should be a close integration of preclinical research with the anticipated clinical trial. Specifically, the preclinical model should incorporate physiologically relevant doses of both the otoprotectant and cisplatin, deliver these agents by the same schedule and route as planned in the clinical trial, and screen for tumor protection using appropriate pediatric cancers. If applicable, screening for tumor protection should include other chemotherapeutic agents that may be included in the planned cisplatin regimen.

## 3 ISSUES IN CLINICAL TRIAL DESIGN AND CONDUCT

For agents demonstrating preclinical activity, a clinical trial is the essential translational step for confirming otoprotection and lack of tumor protection in humans, and for determining the side effect profile and burden of administration. Considerations important in designing an otoprotection clinical trial include the endpoints of interest, outcome assessment, patient cohort characteristics, sample size, and the agent’s mode of delivery. Additionally, the trial must be practical and likely to accrue participants in a timely fashion, a frequent challenge in pediatric supportive oncology research.^41^

### 3.1 Ototoxicity assessment

For clinical trials where prevention or reduction in cisplatin‐induced ototoxicity is the primary endpoint, a measure is needed that is valid, reliable, and sensitive to clinically relevant change. Feasibility for clinical use, suitability for children of varying ages and degrees of illness, and high inter‐rater reliability are important characteristics. Ototoxicity is usually assessed as hearing loss. Although rarely assessed, clinically significant tinnitus often accompanies CIHL, especially among older adolescents and young adults.^10^, ^42^ The standard dictionary for reporting and grading severity of adverse events on National Cancer Institute (NCI)‐sponsored clinical trials is the Common Terminology Criteria for Adverse Event Reporting (CTCAE).^43^ These grading criteria, which are intended to identify harm that requires clinical action (eg, cisplatin dose modification), may not be sufficiently sensitive as an endpoint in a toxicity reduction trial. A clinical trial for an otoprotectant must be able to capture mild, but functionally important, CIHL, particularly in young children.

In clinical trials, audiologic evaluations are the standard hearing assessment for older children and adolescents (Table 1). In younger children, temporary conductive hearing losses can frequently occur during therapy and may be confused with ototoxicity.^44^ Very young, sick, or developmentally limited children may not be able to complete a full evaluation, hindering accurate hearing assessment. Alternative testing modalities, such as auditory brainstem response (ABR), may be needed postoperatively, early in treatment, or in very young children. However, switching assessment modalities between baseline and subsequent time points renders comparison for interval change more challenging.

**Table 1:** Hearing assessments used for ototoxicity monitoring

Test	Test description	Age range	Research applications
Pure tone audiometry	Behavioral measurement of hearing thresholds in decibels (dB) for the speech frequency range (250‐8000 Hz). Testing requires participation and cooperation of the subject. Assessment methods for young children include visual reinforcement audiometry (VRA) and conditioned play audiometry.	8 mo and older	Standard method for hearing measurement, detection of ototoxic damage and identification of communicatively significant hearing loss.
Extended high frequency audiometry	Behavioral measurement of hearing thresholds in decibels (dB) for frequencies above the speech range. Test frequencies include 9000 Hz up to 20 000 Hz. Testing requires participation and cooperation of the subject. Not available at all institutions.	4‐5 y and older	Provides a more sensitive and earlier signal of ototoxic damage because ototoxicity initially occurs at the highest audible frequencies.
Otoacoustic emissions (OAEs)	Objective measurement of cochlear outer hair cell function. Does not require active subject participation. In the presence of normal middle ear function, loss of OAEs suggests outer hair cell damage but additional testing is needed to quantify change in hearing sensitivity. Available at most institutions.	Any age	Typically provides a more sensitive and earlier signal of ototoxic damage. Available at most institutions.
Auditory brainstem response (ABR/BAER)	Objective measurement of neural responses to sound stimuli (auditory evoked potentials). For ototoxicity monitoring, tone burst stimuli are used to estimate hearing thresholds when behavioral audiometry is not possible due to age, development, or medical condition. The subject must be sleeping or lying completely still during testing. Useful for very young, medically debilitated or otherwise uncooperative patient (who may be sedated).	Any age	Standard method for hearing measurement, ototoxicity detection, and identification of communicatively significant hearing loss when behavioral testing is not possible.
Tympanometry	Objective measurement of middle ear pressure and function. Used to determine middle ear status.	Any age	Necessary for valid interpretation of OAEs and to identify conductive middle ear pathology.

Variation among measures of hearing loss hinders comparisons across published clinical trials.^44^, ^45^ More than 8 different criteria have been used to capture and assign hearing loss severity in pediatric cancer trials.^46^ In 2007, COG initiated ACCL05C1, a prospective observational cohort study of children receiving cisplatin whose primary aim was to compare several hearing loss criteria in wide use at the time of study development, specifically, the American Speech‐Language Hearing Association (ASHA),^47^ Brock,^48^ and the CTCAE v 3.0 criteria.^49^ Because raw, individual audiologic results were submitted for central review, it was possible to include the more recent SIOP‐Boston Scale, which was developed in 2012 through international consensus to address limitations posed by the other hearing loss criteria.^50^ The comparative analysis demonstrated that the SIOP‐Boston Scale detected ototoxicity earlier than the other three measures, resulted in the highest percentage of evaluable audiograms, and had an acceptable false‐positive rate. This scale offers advantages that include not requiring a baseline assessment for comparison; being sensitive to CIHL; and aligning with functional patient outcomes.^50^, ^51^

A key finding from ACCL05C1 was that central audiology review proved feasible in the cooperative group setting, as data from 1436 assessments conducted at 53 participating institutions were successfully submitted to the COG coordinating center. Central review improved standardization of grading compared to institutional reporting and provided the opportunity to re‐grade severity by the newer SIOP‐Boston Scale.

### 3.2 Cohort selection and trial feasibility

ACCL0431 was a COG trial designed as a proof‐of‐concept study to determine whether STS prevented CIHL in children.^18^ The cohort selection was intentionally broad to facilitate patient recruitment, but created challenges in interpreting the results of the trial. Participants were 1‐18 years old and newly diagnosed with any cancer for which a cumulative dose of cisplatin ≥200 mg/m^2^ was planned. The principal diagnoses were germ cell tumor, hepatoblastoma, medulloblastoma, neuroblastoma, and osteosarcoma. The rationale for including multiple tumor types was the assumption that diagnosis would not impact the risk of ototoxicity after adjusting for age, cisplatin regimen, and cranial irradiation. Participants were randomized to receive or not receive STS in addition to their disease‐specific cisplatin‐containing regimen. The primary endpoint was development of hearing loss, as defined by ASHA criteria, at 4 weeks following the final dose of cisplatin. Event‐free survival (EFS) and overall survival (OS) were monitored as secondary endpoints. Because the sample size was determined in relation to the primary aim of hearing loss, ACCL0431 had power to detect only large differences in survival.

There were 125 eligible, randomized participants (Table 2). Among the 104 participants evaluable for the primary endpoint, the incidence of CIHL among those who received STS was approximately half that of controls (Table 3). Among all 125 participants in aggregate, there was no statistically significant difference in EFS and OS at a median follow‐up of 3.5 years (Table 3). However, a difference in OS that approached statistical significance favoring the control group prompted an unplanned, post hoc analysis stratified by extent of disease (localized vs disseminated). In that analysis, participants with localized disease demonstrated no survival difference by randomized group, whereas those with disseminated disease demonstrated significantly lower EFS and OS if they received STS (Table 3). Progressive cancer accounted for all but one death. Taken together, these results were definitive for the primary outcome of otoprotection but concerning for the possibility of systemic tumor protection by STS.

**Table 2:** Cohort characteristics of the ACCL0431 study^18^

	Controln (%)	STSn (%)
Age (y)		
<5	22 (34)	22 (36)
5‐9	13 (20)	7 (11)
10‐14	14 (22)	16 (26)
15‐18	15 (23)	16 (26)
Cancer type		
Germ cell tumor	16 (25)	16 (26)
Hepatoblastoma	5 (8)	2 (3)
Medulloblastoma or CNS PNET	14 (22)	12 (20)
Neuroblastoma	12 (19)	14 (23)
Osteosarcoma	15 (23)	14 (23)
Other	2 (3)	3 (5)
Extent of disease^a^		
Localized	38 (59)	39 (64)
Disseminated	26 (41)	21 (34)
Unknown	0	1 (2)

a Determined post hoc.

**Table 3:** Major results of the ACCL0431 study^18^

	Control group % (95% Confidence Interval)	Sodium thiosulfate Group % (95% Confidence Interval)	*P*	Risk ratio (95% Confidence Interval)	*P*
Hearing Loss^a^	56.4 (16.6, 43.3)	28.6 (42.3, 69.7)	.00022^b^	0.31^c^ (0.13‐0.73)	.0036^d^
Survival^e^					
Event‐Free	64 (50, 74)	54 (40, 66)	‐	1.30^f^ (0.75, 2.26)	.36^g^
Overall	87 (76, 93)	70 (56, 80)	‐	2.03^f^ (0.93, 4.44)	.07^g^

a As defined by the American Speech‐Language Hearing Association (ASHA).

b Chi‐square test of proportions.

c Odds ratio.

d Logistic regression test.

e At 3 y, all eligible participants combined.

f Relative hazard ratio.

g Log‐rank test.

The design of ACCL0431 afforded both strengths and limitations. Use of a heterogeneous cohort simultaneously facilitated accrual and provided definitive evidence of otoprotection by STS. On the other hand, heterogeneity of the cohort limited the ability to determine the effect of STS on survival because of the small sample size within each diagnosis type (Table 2), particularly when further divided by extent of disease.

A consideration from the ACCL0431 experience is that heterogeneous diagnoses precluded the ability to consider detailed prognostic information as potential factors in survival. Although cancer types, extent of disease, and age of participants appeared to be balanced in the two randomization groups on ACCL0431, it is possible that within cancer types there was an imbalance of unmeasured biological features that influence outcome and would be incorporated in a more sophisticated risk‐group assignment. Thus, designing a trial to evaluate for an otoprotection signal alone may not provide all the information needed to translate unanticipated results into practice if a heterogeneous cohort is enrolled.

A study design limiting the population to a single cancer type treated with a protocol‐specified chemotherapy regimen would allow cisplatin dose modifications, deletions, and delays to be captured. Yet, many pediatric cancers are rare. Therefore, a conventional noninferiority trial design evaluating an otoprotectant and its potential for tumor protection within a single cancer type would require large sample sizes to exclude small but meaningful decrements in event‐free or overall survival. One such study, a European trial randomizing standard risk hepatoblastoma patients to STS or observation (SIOPEL‐6), has recently been completed.^52^, ^53^

Future trial designs employing methods to assess co‐primary endpoints, for example, otoprotection and progression‐free survival, should be used by building on the lessons of these early efforts and by following FDA guidance for multiple endpoints in clinical trials.^54^ Such designs will require appropriate planning, including sufficiently large sample sizes to examine joint effects in the most promising otoprotection treatment strategies.

### 3.3 Delivery of the otoprotection agent

ACCL0431 addressed the concern about the systemic delivery of both cisplatin and STS using a strategy of time separation of cisplatin and STS. Based on preclinical studies, a 6‐hour interval between completion of cisplatin and administration of STS was specified to ensure preservation of cisplatin anticancer activity.^55^, ^56^ An alternative strategy for avoiding interference with systemic chemotherapy is utilization of chemoprotectants whose activity is anatomically restricted through their pharmacokinetic properties or via loco‐regional administration. An example of the former is mesna, a systemically delivered uroprotectant to prevent hemorrhagic cystitis that is activated only upon excretion via the kidney.^57^, ^58^

In concept, loco‐regional delivery of an otoprotectant by intratympanic injection is associated with minimal systemic absorption and positions the otoprotectant in contact with the round window through which it diffuses into the cochlea.^59^ A loco‐regional approach affords an efficient accrual design and statistical evaluation approach through randomization “by ear” (ie, in a single patient use of one ear for the intervention and the contralateral ear for the control). However, challenges also exist for the intratympanic approach, which include the potential need for procedural sedation, availability of pediatric otolaryngologists, need for coordination of medical and surgical teams, and potential effects on hearing from the intratympanic agent and vehicle.^60^, ^61^

## 4 CONCLUSION

With ACCL0431 providing proof‐of‐principle that CIHL can be prevented, interest in preventing CIHL is growing and new otoprotection agents are in early stages of development. What major insights from ACCL05C1 and ACCL0431 can inform optimal design of future otoprotection trials? From ACCL05C1, we recommend: (1) use of uniform, internationally accepted ototoxicity criteria that are optimal for children (eg, the SIOP‐Boston Scale); and (2) use of central review of raw audiologic data to improve accuracy, reduce bias, and allow use of multiple ototoxicity criteria for comparison between randomized groups. From ACCL0431, we recommend: (1) use of a study cohort that is well‐characterized for disease characteristics and treatment variables to reduce the impact of confounders on both ototoxicity and survival outcomes; and (2) ensuring sufficient statistical methods, power, and sample size to assess multiple primary endpoints and interactions.

In addition, insights reinforced by the COG experience include ensuring that the preclinical studies and clinical trials are exceptionally well integrated with regard to dose, schedule, route, and administration of the otoprotectant and cisplatin, as well as the cancers being studied. Ideally, identification of otoprotectants that are mechanistically distinct from the chemotherapy would minimize concern for inhibition of chemotherapy effects. Alternatively, loco‐regional administration of otoprotectants by intratympanic injection offers similar theoretical advantages. Clearly, more stringent requirements for the study population may require longer recruitment periods and may limit feasibility and generalizability. Thus, a balance of the study cohort and the specific endpoints is a consideration for each trial design. Published final results of SIOPEL‐6, which used an alternative study design, will contribute to our understanding of optimal approaches for pediatric otoprotection trials, as well as the emerging discussion about a potential clinical role for STS and the potential generalizability of its use to prevent ototoxicity beyond hepatoblastoma.^53^ As well, there may be a need for future discussion about what might constitute an appropriate control arm in the future randomized clinical trials of new otoprotectants.

Regardless of study design, other aspects of ototoxicity, including tinnitus, reduced speech comprehension in noisy environments, and impact of hearing loss on daily function and quality of life, are important outcomes. We recommend including a more comprehensive set of measures for these in the future clinical trials, particularly for older children, adolescents, and young adults who can provide patient‐reported outcomes. Additional studies may further elucidate novel agents and approaches to reduce or prevent ototoxicity. The National Institutes of Health (NIH) released several funding announcements (Table 4) specifically to highlight the need to develop preclinical models to study the effects of anticancer agents and regimens on normal tissue and on the development of late adverse effects, and to identify patients at highest risk of treatment‐related complications.

**Table 4:** NIH funding opportunity announcements

Funding opportunity announcement	Number	Link
PPQ‐6: How can mouse or other preclinical models be used to study how standard of care and investigational therapies affect normal tissue and lead to adverse events later in life?	PAR‐16‐217	Research Answers to NCI’s Pediatric Provocative Questions (R21) https://grants.nih.gov/grants/guide/pa-files/PAR-16-217.html
PPQ‐7: How can prediction models be developed and used to identify patients at highest risk of treatment‐related complications?	PAR‐16‐218	NCI Research Answers to NCI’s Pediatric Provocative Questions (R01) https://grants.nih.gov/grants/guide/pa-files/PAR-16-218.html
National Cancer Institute (NCI) Mechanisms of Cancer and Treatment‐related Symptoms and Toxicities (R21)	PA‐16‐258	https://grants.nih.gov/grants/guide/pa-files/PA-16-258.html
National Institutes of Health (NIH) Serious Adverse Drug Reaction Research (R01/R21)	PAR‐16‐275 and PAR‐16‐274	https://grants.nih.gov/grants/guide/pa-files/PAR-16-275.html and https://grants.nih.gov/grants/guide/pa-files/PAR-16-274.html

The successes of ACCL0431 and ACCL05C1 are but the beginning of a new era of otoprotection research for children facing the prospect of developing CIHL. These studies have contributed to a paradigm shift in pediatric oncology that no longer considers CIHL as acceptable and demands continued research to expand the available options for effective otoprotection and refine the ways in which these are applied.

## CONFLICT OF INTEREST

David R. Freyer is an uncompensated scientific advisor for *Otonomy*. A. Lindsay Frazier serves on the Clinical Advisory Board of *Decibel Therapeutics*
